# Dynamic Changes in Metabolites and Transformation Pathways in Diqing Tibetan Pig Hams During Fermentation Determined by Widely Targeted Metabolomic Analysis

**DOI:** 10.3390/foods14142468

**Published:** 2025-07-14

**Authors:** Dan Jia, Siqi Jin, Jin Zhang, Shuyuan Luo, Xinpeng Li, Siew-Young Quek, Xinxing Dong, Dawei Yan

**Affiliations:** 1 College of Animal Science and Technology, Yunnan Agricultural University, Kunming 650201, China; jiadan@ynau.edu.cn (D.J.); jsq34561234@163.com (S.J.); 15118416@njau.edu.cn (S.L.); lixinpeng9812@163.com (X.L.); 2012045@ynau.edu.cn (X.D.); 2 State Key Laboratory for Managing Biotic and Chemical Threats to the Quality and Safety of Agro-Products, Institute of Food Science, Zhejiang Academy of Agricultural Sciences, Hangzhou 310021, China; zhangjin@zaas.ac.cn; 3 Food Science, School of Chemical Sciences, The University of Auckland, Auckland 1010, New Zealand; sy.quek@auckland.ac.nz

**Keywords:** metabolic pathways, Diqing Tibetan pig hams, fermentation, KEGG pathway analysis, widely targeted metabolome

## Abstract

This study investigated the metabolite dynamic changes and transformation pathways in Diqing Tibetan pig (DTP) hams during fermentation (0, 30, 90, 180, 360, 540 d) by widely targeted metabolomics. A total of 873 metabolites in 17 subclasses were detected, with significant changes in 448 metabolites. Additionally, 65 key metabolites were found to be involved in the top 10 pathways, with the top pathways for metabolite markers in mature hams including protein metabolism (2-oxocarboxylic acid metabolism, tryptophan metabolism and amino acid biosynthesis) and lipid metabolism (unsaturated fatty acid biosynthesis and linoleic acid metabolism). Overall, the unique DTP ham taste, flavor, and nutritional value may be contributed to by the significant accumulation of essential amino acids, MSG-like amino acids, free fatty acids (arachidonic acid, docosahexaenoic acid, and eicosapentaenoic acid), citric acid, oxaloacetic acid, succinic acid, and vitamin B. This study facilitates a comprehensive understanding of metabolic profiling and the transformation pathways of DTP hams during fermentation, providing novel insights into the biochemical mechanisms underlying traditional Tibetan pig hams, bridging traditional knowledge with modern omics technologies.

## 1. Introduction

Diqing Tibetan pigs, an ideal animal for manufacturing top quality dry-cured hams, are primarily distributed in Xianggelila County, Yunan, China (a 3500–4500 m high-altitude area). In our previous study, we elucidated the changes in Diqing Tibetan pig (DTP) ham physicochemical and volatile flavor compounds during fermentation [1]. However, the dynamic changes in metabolites in DTP hams and their transformation pathways during fermentation remained to be investigated.

Non-volatile metabolites, including amino acids, organic acids, lipids, nucleotides, and their derivatives, have been reported to exert a crucial role in the formation of the flavor and taste of hams [2]. Taste, as a critical indicator of meat products, is a decisive factor in the overall quality of dry-cured hams, so many reports have classified non-volatile metabolites as the taste compounds with the most intense influence on taste development in dry-cured hams, including free amino acids, organic acids, nucleotides, and small peptides [3]. Additionally, the flavor of dry-cured hams has been shown to be due to a complex process of various chemical reactions, including protein hydrolysis, lipid oxidation, and sugar fragmentation related to the Maillard reaction, leading to the generation of acids, esters, ketones, alcohols, hydrocarbons, and other flavor substances [4].

In recent studies on muscle metabolism and meat quality, metabolomics has been widely applied to analyze variations in small molecular substances, such as amino acids, sugars, fatty acids, organic acids, and other compounds which might together influence the flavor and taste of meat and meat products [5]. Based on specific analytical objectives, metabolomics can be classified as a combination of non-targeted and targeted metabolomic technologies, and it is commonly used to examine non-volatile metabolite dynamic changes [6]. For instance, Liu et al. [7] used non-targeted metabolomics to reveal variations in small molecular substances in duck meat preserved for different periods of time. They found that with increasing preservation time, the duck meat showed an increase in the content of nitrogen-containing small molecular compounds, organic acids, and carbohydrates, and that the nucleotide decomposition process was strengthened. Additionally, non-targeted metabolomics was adopted to reveal the metabolites of Zhenba bacon produced from different pig parts [4]. Moreover, non-targeted metabolomics was used to evaluate the effects of partial NaCl substitution by KCl on small molecular metabolites of Xuanwei ham [8]. Meanwhile, targeted metabolomics was used to identify metabolite biomarkers by the quantitative analysis of fresh and *Salmonella enteritidis*-contaminated chickens [9]. Additionally, through targeted metabolomics analysis, 18 hydrophilic metabolites were identified to be affected by castration in the psoas major muscles of castrated lambs [10]. In summary, non-targeted metabolomics is high in coverage but limited in sensitivity, thus suffering the defects of false positive results, while targeted metabolomics is low in coverage, allowing the use of a single-run approach to analyze only one class of non-volatile metabolites [6]. Therefore, widely targeted metabolomics shared these mutual advantages, enabling broad coverage, high throughput, and superior sensitivity, leading to its successful application in food science research, such as in peanut roasting [11], the analysis of green tea at different stages [12], and key loquat component identification [13]. In meat research, widely targeted metabolomics had been applied to investigate metabolite dynamic variations and transformation pathways in Mongolian sheep during early postmortem chilled aging [14]. However, to the best of our knowledge, no researchers have ever used widely targeted metabolomics to explore changes in DTP ham metabolites and their transformation pathways, which might determine ham quality during fermentation. Therefore, this study aimed to fill this research gap by using widely targeted metabolomics to evaluate dynamic changes in DTP ham metabolites during fermentation, including metabolic markers and metabolic pathways. The results facilitate a better understanding of variations in metabolism and key metabolic pathways associated with the quality of hams during fermentation, laying a theoretical foundation for improving industrial DTP ham quality.

## 2. Material and Methods

### 2.1. Ethics Statement

All animal handling procedures and sample collection methods in this study were approved by the Life Science Ethics Committee of Yunnan Agricultural University (Approval No. 202205004) and were conducted in accordance with local Chinese laws and institutional animal care guidelines.

### 2.2. Materials and Sample Collection

In this study, all 15 DTPs were grown under the same rearing conditions. They were uniformly slaughtered in mid-December according to standard slaughtering procedures. The pigs were fasted for 24 h before slaughter, provided with free drinking water, and were not whipped or chased before slaughter to avoid interfering with the meat slaughtering experiments conducted in the slaughterhouse. All pigs were stunned electrically and then slaughtered by rapid cardiac bloodletting, followed by the fine division of the carcass of each pig. Hams were prepared from 30 fresh DTP hind legs. Specifically, the hams were treated with well salt (3.5 g/kg green ham) (0 d) and then stored for 7 d in a workshop (85–90% relative humidity (RH), 0–5 °C), followed by placing the salted legs in a balance room for 30 days (70–75% RH, 7–8 °C) (30 d). Finally, the drying hams were stored for ripening at 65% RH and 0–15 °C, with 90, 180, 360, and 540 d hams collected during this ripening process. At each sampling period, biceps femoris muscle samples were collected as mixed samples from the corresponding groups (0, 30, 90, 180, 360, and 540 d) of ham samples, followed by storage at −80 °C for further use.

### 2.3. Metabolomic Profiling

Sample preparation, the analysis of extraction samples, and the detection and quantification of different metabolites were performed according to the description by Zhang et al. [14].

#### 2.3.1. Preparation of Samples for Metabolomic Profiling Analysis

Metabolomic profiling was performed for biceps femoris muscle samples as described above. Briefly, the samples (20 mg) were mixed with 400 μL of ice-cold methanol/water (70% *v*/*v*), and the final methanol internal standard was 1 μg/mL, followed by 3 min of homogenization at 30 Hz, 1 min of vortexing, and 10 min of centrifugation at 12,000× *g* and 4 °C (Eppendorf, 5424R, Hamburg, Germany). The filtrate was stored at −20 °C for 30 min. Finally, the stored filtrate was centrifuged for 3 min at 4 °C and 12,000× *g*, and 200 μL of supernatant was transferred to a vial and maintained at −80 °C for further analysis (Thermo Fisher, 907, Waltham, MA, USA). Each sample was tested with 6 replicates.

#### 2.3.2. T3 UPLC Conditions

Using the method of Zhang et al. [14], extraction samples were analyzed using an ultraperformance liquid chromatography (UPLC) instrument (ExionLC AD) with ACQUITY HSS T3 C18 (2.1 × 100 mm,1.8 µm; Waters, Milford, MA, USA) plus a QTRAP^®^ MS/MS system (Applied Biosystems, Foster City, CA, USA). Metabolites were eluted stepwise with solvent A (0.1% formic acid in water) and solvent B (0.1% formic acid in acetonitrile) at 0.4 mL/min (0 min 5% B, 11.0 min 90% B, 12.0 min 90% B, 12.1 min 5% B, 14.0 min 5% B), a 40 °C column temperature, and a 2 μL injection volume.

#### 2.3.3. ESI-QTRAP-MS/MS

A triple quadrupole linear ion trap mass spectrometer (QTRAP) was used to obtain triple quadrupole (QQQ) and linear ion trap (LIT) scans. The effluent quality was analyzed using Analyst 1.6.3 software in both ion modes on the QTRAP^®^ LC-MS/MS system with an electron spray ionization (ESI) Turbo Ion-Spray interface (AB Sciex, Framingham, MA, USA). The ESI conditions consisted of a 500 °C temperature; collision gas set to high; 55, 60, and 25 psi ion source gas I, gas II, and gas curtain pressures; and 5500 V (positive mode) or −4500 V (negative mode) ion spray voltages. Additionally, 10 and 100 mmol/L polypropylene glycol solutions were used for instrument adjustment and mass calibration in QQQ and LIT modes, respectively. Quality control (QC) samples were prepared by mixing the sample extracts, and for every ten test samples, one QC sample was inserted to monitor a fixed number of experiments for a repeatability analysis [14].

#### 2.3.4. Qualitative and Quantitative Analysis

After metabolomics analysis, all the raw data were imported into Analyst 1.6.3 software. The qualitative analysis of metabolites was implemented based on their retention time, precursor ion pair information, and secondary spectrum data. Multiple reaction monitoring analysis was used to quantify the metabolites by triple quadrupole mass spectrometry. With the criteria of an area > 1000, signal-to-noise ratio > 3, and CV < 50%, the metabolites in each sample were identified. All of the metabolites were recorded, and their quantitative indices were standardized using the range method in R software after the screening process.

### 2.4. Statistical Analysis

During metabolomic data analysis, ANOVA applicability was determined first by analyzing data variance homogeneity and normal distribution assumption. The R package MetaboAnalystR (v. 1.0.1) was used for orthogonal partial least squares discriminant analysis (OPLS-DA), and the prcomp function in the R package was used to perform principal component analysis (PCA), with metabolite data being log_2_ transformed (log_2_) and mean-centering being performed prior to OPLS-DA. From the OPLS-DA results, variable importance of projection (VIP) results were obtained in the form of score and permutation plots. Finally, differential metabolites with significant changes were screened based on VIP ≥ 1. The VennDiagram package was also used to generate metabolite Venn diagrams to reflect the variations in metabolites in each group. The differential metabolites were analyzed based on the Kyoto Encyclopedia of Genes and Genomes (KEGG) database, and the pathways able to significantly regulate the metabolites were used for further enrichment analysis, with their significance level determined by *p* values. Cytoscape software (v. 1.10.0) was used to draw the interactive networks and pathways of the metabolites.

## 3. Results and Discussion

### 3.1. Full Mass Spectrometric Analysis of Metabolite Dynamic Changes During Diqing Tibetan Pig Ham Fermentation

**Figure 1:**
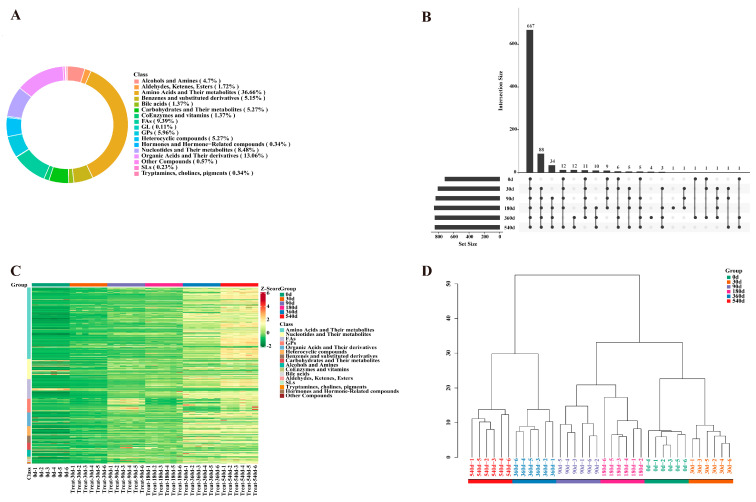
Metabolites of Diqing Tibetan pig hams during fermentation. (**A**) Ring chart of proportion of all metabolite categories. (**B**) Advanced Venn diagram of metabolites at various fermentation stages (0 d, 30 d, 90 d, 180 d, 360 d, and 540 d). Black dots, overlapping groups; intersection size, number of cross metabolites; set size, number of metabolites in each group. (**C**) Heat map for levels of metabolites during fermentation. (**D**) Sample hierarchical clustering tree for metabolites during fermentation. FAs: fatty acyls; GPs: glycerophospholipids; SLs: sphingolipids; Others or Other Compounds: abietic acid, betaine, butenoyl-PAF, kojibiose, and tetramethylthiuram disulfide.

In this study, the metabolomic analysis of DTP ham samples collected at 0, 30, 90, 180, 360, and 540 d identified a total of 873 metabolites, including amino acids and their metabolites (36.66%); organic acids and their derivatives (13.06%); fatty acyls (FAs) (9.39%); nucleotides and their metabolites (8.48%); glycerophospholipids (GPs) (5.96%); carbohydrates and their metabolites (5.27%); heterocyclic compounds (5.27%); benzenes and substituted derivatives (5.15%); alcohols and amines (4.70%); aldehydes, ketones, and esters (1.72%); coenzymes and vitamins (1.37%); etc. (Figure 1A). In Figure 1B, the advanced Venn diagram displays the common and unique DTP ham metabolites at different fermentation stages, with the number of metabolites increasing with fermentation time. In Figure 1C, the metabolite classification heat maps more vividly display the variations in total metabolites. During fermentation, except for a decrease in some FAs and a few metabolites, the overall DTP ham metabolites showed a significant increase during 360–540 d fermentation. In the hierarchical clustering tree of Figure 1D, 0–30 d DTP hams can be seen to form a cluster, as well as a 90–180 d cluster, leading to the formation of one large branch. Moreover, 360–540 d hams were closely clustered and formed independent clusters, indicating that metabolite profiles were different between 360–540 d and 0–180 d hams. These results were consistent with our previous report that 360–540 d DTP hams tended to be mature, having a flavor and taste different from that of 0–180 d hams [1]. 

### 3.2. Identification of Key Diqing Tibetan Pig Ham Metabolites During Fermentation

**Figure 2:**
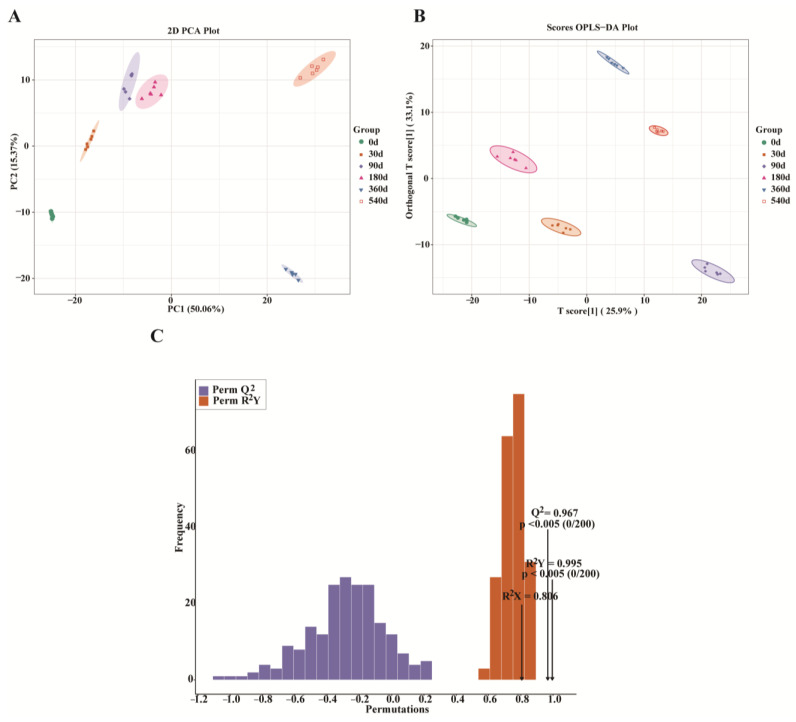
Establishment and validation of orthogonal partial least squares discriminant analysis (OPLS-DA) model. (**A**) Plot of PCA scores for different fermentation times. (**B**) Plot of PLS-DA scores for different fermentation times. (**C**) OPLS-DA model validation, with R^2^ X = 0.806, R^2^ Y = 0.995, and Q^2^ = 0.967 in permutation test.

Changes in differential metabolites during DTP ham fermentation were further investigated by PCA and OPLS-DA. As shown in Figure 2A, 50.06% and 15.37% of total variance could be explained by the first and second principal components, respectively. There were clear separations between the samples collected at different fermentation periods, with 30–180 d hams far away from the 0 d ham samples, and 360 d and 540 d hams significantly different from 0–180 d hams, indicating the greatest changes in flavor composition for 360–540 d DTP hams versus 0–180 d ham samples. In Figure 2B, OPLS-DA score analysis visually distinguishes DTP hams collected at 0, 30, 90, 180, 360, and 540 d. Specifically, the OPLS-DA score plotting and model validation were performed using six fermentation periods as dependent variables and 873 metabolites as independent variables (Figure 2B,C), where the first two principal components were seen to explain 25.90% and 33.10% of total variance and there was a clear separation between different fermentation periods. In Figure 2C, the evaluation parameters (Q^2^ = 0.967, *p* < 0.005) and (R^2^ Y = 0.995, *p* < 0.005) indicate the high reliability of the OPLS-DA model used in this study. Moreover, the VIP values were used to determine the discriminating metabolites among DTP hams during fermentation. Specifically, 448 differential metabolites were identified with VIP ≥ 1 by combining univariate and multivariate statistical analysis results (Table S1).

**Figure 3:**
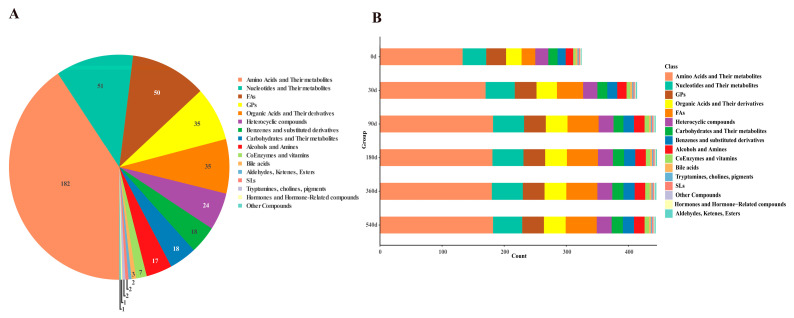
Differential metabolites in Diqing Tibetan pig hams during fermentation. (**A**) Pie chart of classification of 448 differential metabolites. (**B**) Bar graph of number and classification of differential metabolites at each fermentation stage. FAs: fatty acyls; GPs: glycerophospholipids; SLs: sphingolipids; Others: abietic acid, betaine, butenoyl-PAF, kojibiose, and tetramethylthiuram disulfide.

These 448 differential metabolites are derived from 16 types of substances, including 182 amino acids and metabolites; 51 nucleotides and metabolites; 50 FAs; 35 GPs; 35 organic acids and derivatives; 24 heterocyclic compounds; 18 benzenes and substituted derivatives; 18 carbohydrates and metabolites; 17 alcohols and amines; 7 coenzymes and vitamins; 3 bile acids; 2 aldehydes, ketones, and esters; 2 tryptamines, cholines, and pigments; 1 hormone or hormone-related compound; and 1 other (Figure 3A). Among them, the main differential DTP ham metabolites during fermentation were amino acids and their metabolites, organic acids and their derivatives, nucleotides and their metabolites, FAs, and GPs.

Amino acids and nucleotides have been reported to be essential contributors to taste formation, and the flavor compounds produced by FA oxidation can greatly enhance the flavor of dry-cured ham, i.e., the primary products of lipid oxidation can further react with free amino acids through the Maillard reaction to generate more flavor compounds [2]. This suggests that the taste and flavor of DTP hams could significantly improve during fermentation.

The DTP hams showed significant changes in the number of metabolites during fermentation (Figure 3B). With the increase in fermentation time, a number of metabolites were generated, leading to an increase in total metabolite number and especially a significant upward trend in the levels of amino acids and their metabolites, nucleotides and their metabolites, and FAs during DTP ham fermentation. Generally, meat quality is based on proteins, lipids, peptides, and small molecular metabolites, and fermentation can promote increases in the content of amino acids, lipids, and nucleotides and their derivatives in DTP hams, enabling DTP hams to be more abundant in taste and flavor after fermentation.

**Figure 4:**
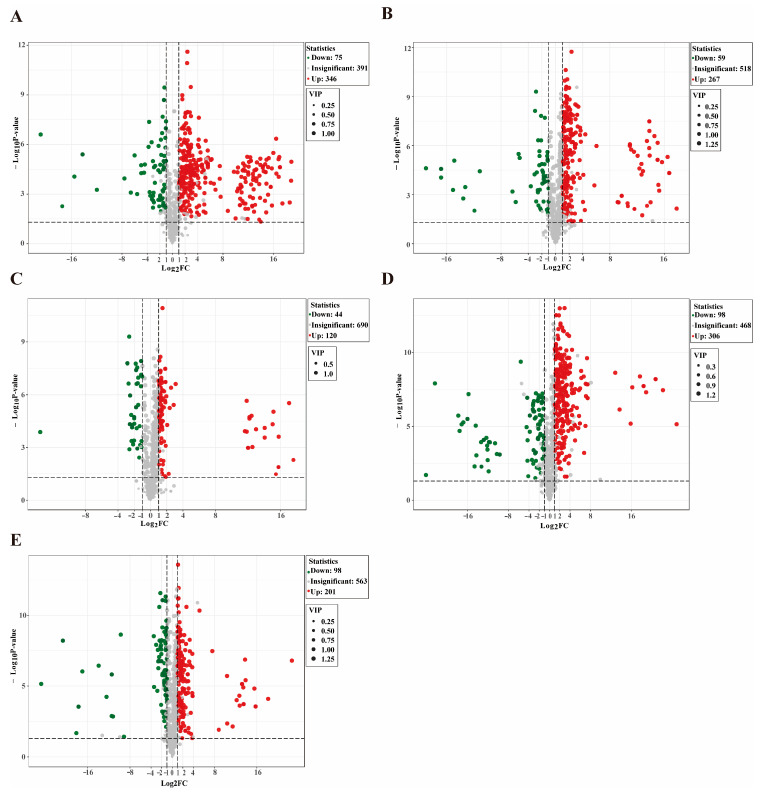
Volcano plots of Diqing Tibetan pig hams during fermentation: 0 d vs. 30 d (**A**); 30 d vs. 90 d (**B**); 90 d vs. 180 d (**C**); 180 d vs. 360 d (**D**); 360 d vs. 540 d (**E**).

Figure 4 volcano plots for the metabolite expression differences between DTP hams at two successive fermentation stages, where the blue, red and gray dots represent the downregulation of, upregulation of, and no significant changes in the expression of the differential metabolites, with each point representing a metabolite and statistical significance being visualized. The abscissa and ordinate represent the log_2_ fold change (log_2_FC) and FDR-adjusted *p*-value (*p*-value, VIP), respectively. A greater absolute abscissa value indicates a greater log_2_FC in the expression levels between two samples, while a greater ordinate value represents a more significantly differential expression, indicating a higher reliability of the screened metabolites. As shown in Figure 4, 346 metabolites were upregulated and 75 downregulated in the DTP hams from 0 d to 30 d (Figure 4A), 271 upregulated and 59 downregulated from 30 d to 90 d (Figure 4B), 122 upregulated and 44 downregulated from 90 d to 180 d (Figure 4C), 306 upregulated and 98 downregulated from 180 d to 360 d (Figure 4D), and 201 upregulated and 98 downregulated from 360 d to 540 d (Figure 4E). The metabolites showed more drastic changes in the earlier and aged stages of fermentation, consistent with the greater distances between the 0–90 d and 360–540 d hams in the PCA score plot (Figure 2A).

### 3.3. Metabolic Evolution and Transformation Pathways of Characteristic Metabolites During Diqing Tibetan Pig Ham Fermentation

For the 448 differential metabolites, their related pathways during DTP ham fermentation were explored by KEGG analysis (Supplementary Table S1). A total of 152 pathways in six modules were identified: metabolism (74), organismal systems (41), human diseases (20), environmental information processing (12), cellular processes (3), and genetic information processing (2) (Figure S1).

**Figure 5:**
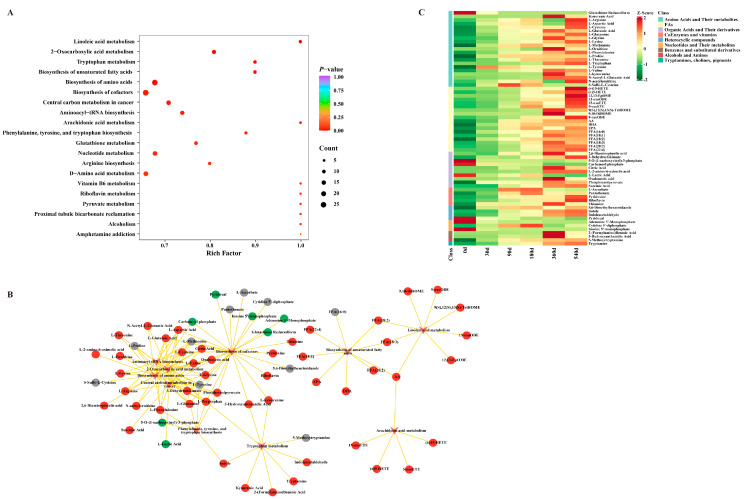
The evolution track of the annotation of differential metabolites during fermentation in Diqing Tibetan pig hams. (**A**) The top20 KEGG enrichment pathways for differential metabolites. (**B**) Network diagrams of the top 10 KEGG enrichment pathways with the annotation of the differential metabolites and red, green, and gray dots for the upregulation of, downregulation of, and no significant change in 360–540 d Diqing Tibetan pig hams, respectively. (**C**) A heat map of the annotated differential metabolites in the top 10 KEGG enrichment pathways. FAs: fatty acyls.

Figure 5 displays the top 20 pathways, where the horizontal and vertical coordinates indicate their respective Rich Factor and name (sorted by *p*-value), and the dot color represents the *p*-value size, with redder colors for more significant enrichments. The dot size shows the number of enriched differential metabolites. The top 10 pathways were amino acid biosynthesis; phenylalanine, tyrosine and tryptophan biosynthesis; arachidonic acid metabolism; linoleic acid metabolism; 2-oxocarboxylic acid metabolism; tryptophan metabolism; unsaturated fatty acid biosynthesis; cofactor biosynthesis; and central carbon metabolism in cancer. Aminoacyl-tRNA biosynthesis, along with linoleic acid metabolism, unsaturated fatty acid biosynthesis, and arachidonic acid metabolism, was relatively independent of the other pathways.

The top 10 pathways and the annotated metabolites were visualized using a network diagram (Figure 5B) to display the transformation of the metabolites. Additionally, a heat map was drawn to show the dynamic changes in annotated metabolites during fermentation (Figure 5C). KEGG enrichment analysis revealed the metabolism of 65 compounds during DTP ham fermentation in the top 10 pathways, including 21 amino acids and their metabolites, 18 FAs, 10 organic acids and their derivatives, 5 coenzymes and vitamins, 4 heterocyclic compounds, 3 nucleotides and their metabolites, 2 benzenes and substituted derivatives, 1 alcohol or amine, and 1 tryptamine, choline, or pigment.

In the present research, the most abundant key metabolites were amino acids and their metabolites, which increased significantly during fermentation in DTP hams. Small molecular peptides and free amino acids have been evidenced to be the main substances with significant contributions to the taste of dry-cured hams [15]. In this study, amino acids and their metabolites showed a similar trend to that shown in Liu et al. [16], who found the generation of a large quantity of amino acids and their derivatives during ham maturation stage, which might greatly affect ham flavor. One of the major contributors to texture transformation and flavor formation is protein hydrolysis, whose effect depends on the activity of lysosomal endopeptidases, exopeptidases, and proteinases. As the end products of protein hydrolysis, small peptides and free amino acids are then subjected to Strecker degradation and deamination for further generation of aromatic aldehydes or linear-chained alcohols [17]. This indicates that the discrepancy in amino acids and their derivatives in the final products might have an important impact on the taste and flavor of DTP hams.

In this study, a large quantity of small peptides and free amino acids were accumulated during the DTP ham aging process, probably due to meat protein degradation, forming a complex mixture of free amino acids during ripening. Excluding glutathione reduced form, other amino acid metabolites accumulated during fermentation, with the highest accumulation of L-tyrosine at 30 d, S-sulfo-L-cysteine at 90 d, and L-methionine at 180 d. At 360 d, the key metabolites were found to be L-glutamic acid, kynurenic acid, L-glycine, L-ornithnine, L-valine, and L-kynurenine, in contrast to the key metabolites at 540 d, which were N-acetylornithine, L-arginine, L-aspartic acid, L-cysteine, L-glutamine, L-lysine, L-phenylalanine, L-threonine, and L-tryptophan. According to the metabolomics data, matured hams showed a significant accumulation of essential amino acids (L-phenylalanine, L-lysine, L-threonine, L-tryptophan, and L-valine), indicating that fermentation enhanced the nutritional value of DTP hams. Figure 5B showed that free amino acids were probably involved in the pathways of phenylalanine, tyrosine, and tryptophan biosynthesis, 2-oxocarboxylic acid metabolism, amino acid biosynthesis, cofactor biosynthesis, central carbon metabolism in cancer, and aminoacyl-tRNA biosynthesis for metabolic transformation in DTP ham during fermentation. During the Iberian ham ripening process, researchers found a linear increase in L-valine, L-lysine, L-leucine and L-isoleucine [18]. In the current study, L-lysine and L-valine were found in mature DTP hams, while no L-leucine (with a bitter taste) or L-isoleucine were detected. As reported by Pérez-Santaescolástica et al. [19], free amino acids can be grouped according to their taste characteristics (sweet, sour, bitter, monosodium glutamate (MSG)-like). In this study, the matured DTP hams showed accumulation of MSG-like amino acids (L-glutamic acid and L-aspartic), sweet amino acids (L-glycine, L-threonine, L-proline, L-lysine, and L-glutamine), and bitter amino acids (L-arginine, L-phenylalanine, and L-valine), which might give the DTP hams a unique taste. Branched-chain amino acids, such as valine leucine and isoleucine, were shown to mainly participate in the formation of branched aldehyde compounds by Strecker degradation, and they were usually detected in longer-aged hams. In this study, aside from leucine and isoleucine, valine was accumulated in DTP hams at the highest level at 360 d; moreover, 2-methyl-2-propenal was at its highest level in the 360 d DTP hams, and 3-methyl-butenal was highest in the 540 d DTP hams, which might be due to the degradation of isoleucine, leucine, and valine [1]. L-cysteine and L-methionine, as precursors of sulfur-containing volatiles, are important precursors of meat flavor volatiles. In this study, L-cysteine levels increased significantly with fermentation time in DTP hams, while L-methionine levels increased in 30–180 d DTP hams, decreased at 360 d, and then increased at 540 d in DTP hams. In our previous studies, 3-(methylthio) propanal (which has a very low threshold value and smells like soy sauce) was found to probably be derived from cysteine and methionine in DTP hams [1]. It is worth noting that these flavor substances can be mixed with other flavor substances to form the unique flavor of DTP hams. Accordingly, in addition to the individual effect of each amino acid on taste, DTP ham’s overall flavor can also be influenced by the combination of these precursors.

As shown in Figure 5C, 18 FAs were detected in the DTP hams. The FA distribution and concentration are closely associated with meat texture, flavor, and nutritional value. Essential fatty acids, including linoleic acid (FFA 18:2) and alpha-linolenic acid (FFA 18:3), cannot be synthesized in humans and animals; furthermore, they are also precursors for the synthesis of long-chain n-3 fatty acids, including eicosapentaenoic acid (EPA, 20:5) and docosahexaenoic acid (DHA, 22:6), and long-chain n-6 fatty acids, such as arachidonic acid (AA, 20:4). EPA and DHA have been demonstrated to the posterior of, or as derivatives of, alpha-linolenic acid, which serves as their precursor or matrix [20]. In the present study, these fatty acid metabolites displayed an uptrend in abundance during fermentation in mature DTP ham, particularly in the relative content of monounsaturated fatty acids (MUPAs) and polyunsaturated fatty acids (PUFAs), with the highest level of alpha-linolenic acid (FFA 18:3) and oleic acid (FFA 18:1) found in 360 d DTP hams, while the highest content of AA, EPA, FFA (18:2), eicosadienoic acid (FFA 20:2), and docosatetraenoic acid (FFA 22:4) were found in 540 d DTP hams (Figure 5C). Many studies have shown that oleic acid (FFA 18:1) can reduce blood pressure and plasma cholesterol [21]. Additionally, DHA and EPA could exert positive impacts on human nutrition and health, supporting the optimal growth and development of infant brains [22]. Therefore, the enrichment of PUFA and MUFA content can be considered as a contributor to DTP ham nutritional value during fermentation. Additionally, EPA and DHA can not only enhance the meat’s sweetness and umami taste, but can also mask its sourness and bitterness simultaneously [23].

Fatty acid metabolites are primarily formed through fat hydrolysis under the catalysis of lipase. During the ripening process of dry-cured ham, triglycerides and phospholipids are hydrolyzed by lipases and phospholipases, respectively, which are reportedly associated with free fatty acid accumulation and volatile flavor formation. The volatile flavor compounds produced by lipid oxidation play an important role in the odor and aroma of dry-cured ham because of their low threshold. Generally, the oxidation of fatty acids during ham ripening is mainly due to autoxidation affected by PUFAs, resulting in the accumulation of oxidation metabolites such as aldehydes, ketones, and alcohols, which participate in the Maillard reaction, producing heterocyclic compounds to intensify the flavor of aged hams [24]. Free amino acids and fatty acid metabolites displayed abundant accumulation in mature DTP hams, suggesting the co-occurrence of protein degradation and fat hydrolysis in the later aging stages. Pathway analysis also revealed an important role of lipid metabolism during the DTP ham ripening period, mainly involving arachidonic acid metabolism, linoleic acid metabolism, and unsaturated fatty acid biosynthesis, which were relatively independent of the other pathways. The top five pathways included protein metabolism (amino acid biosynthesis, 2-oxocarboxylic acid metabolism, and tryptophan metabolism) and lipid metabolism (linoleic acid metabolism and unsaturated fatty acid biosynthesis), which were shown to be the major pathways performing important functions in mature DTP hams. Combined with our previous results, the contents of volatile flavor compounds were higher in mature DTP hams, with more significant changes found in 360–540 d DTP hams. Specifically, the decrease in DHA and alpha-linolenic acid (FFA 18:3) at 540 d can be attributed to PUFA oxidative degradation to volatile flavor components, such as straight-chain aldehydes or methyl ketones. In our previous study, 2-butanone was found to increase in 540 d DTP hams, probably due to DHA and alpha-linolenic acid (FFA 18:3) degradation [1]. AA is a linoleic acid metabolite; hexanal, which has a relatively low threshold value and a grassy and fatty flavor, is mainly generated from AA automatic oxidation; heptanal, nonanal, and octanal are derived from oleic acid (FFA 18:1) oxidation [25]. Moreover, DTP hams showed the highest level of oleic acid (FFA 18:1) at 360 d and AA at 540 d. Combined with our previous study, the increase in hexanal during fermentation can be attributed to AA oxidation, while the highest contents of octanal (meat-like) and nonanal (fatty) occurring at 180 d might be caused by oleic acid oxidation [1].

9(S), 12(S), 9,10-DiHOME, and 13(S)-TriHOME showed their highest levels in 360 d DTP ham, while (±)15-HETE, (±)5-HETE, 12,13-EpOME, 13-oxoODE, 9-oxoODE, 15-oxoETE, and 5-oxoETE had the highest levels in 540 d DTP ham. 15-oxoETE is a major AA metabolite generated via the lipoxygenase pathway. As reported by Li et al. [26], the fatty acid precursor for (±)15-HETE formation is almost exclusively arachidonic acid added externally, as evidenced by deuterated arachidonic acid incubation experiments. In the present study, AA, 15-oxoETE, 5-oxoETE, (±)15-HETE, and (±)5-HETE were revealed to be involved in arachidonic acid metabolism.

Ten organic acids and their derivatives were deemed as differential metabolites during DTP ham fermentation. The 0 d ham showed the highest content of 5-O-(1-carboxyvinyl)-3-phosphate, carbamoyl phosphate, and L-lactic acid, which decreased along with fermentation time. 2,6-diaminopimelic acid, citric acid, oxaloacetic acid, and phosphoenolpyruvate had the highest content in the 360 d ham, while the 540 d ham exhibited the highest content of 3-dehydroshikimate and succinic acid. L-2-amino-6-oximelic reached its highest levels in 360–540 d hams. According to Zhuang et al. [27], carbamoyl phosphate can be further degraded into ammonia and CO_2_ to produce energy. During fermentation, L-lactic acid levels decreased, probably because the microbiota, including some lactic acid bacteria, can use lactic acid as a substrate under anaerobic conditions [28]. Under the action of transaminase, aspartic acid was transformed into oxaloacetic acid, which was further converted to ketones such as n-butanol and 2-butanone. These results are consistent with our previous publication, which found that the highest content of 2-butanone in 540 d DTP hams might be attributed to the decrease in oxaloacetic acid [1]. Furthermore, the content of 3-dehydroshikimate, an important intermediate product from aromatic amino acid biosynthesis, showed a gradual increase with amino acid metabolism during fermentation. Similar results were also found in yak meat during oxidation [25].

Organic acids also perform a crucial role in promoting taste-related compounds. For example, in boneless dry-cured ham, succinic acid has been found to be a taste-active compound, and a unique umami taste can be generated by the combination of citric, lactic, and succinic acids [29]. In the present study, succinic acid increased along with fermentation time, agreeing with a previous report that the succinic acid content increased with the extension of processing years in Nuodeng hams [30].

During DTP ham fermentation, the levels of five coenzymes and vitamins increased, with the highest levels of L-ascorbate and pantothenate (vitamin B5) being at 180 d, thiamine at 360 d, and pyridoxine and riboflavin at 540 d. As a key precursor for coenzyme A (CoA) biosynthesis, pantothenate performs a significant role in amino acid and lipid metabolism. Meat and meat products have been shown as good sources of vitamin B in previous studies on raw meat and different pork products, including dry-cured hams [31]. Li et al. [32] reported that sulfur-containing volatile compounds were mainly generated from sulfur-containing amino acids and thiamine in meat products, and some volatile compounds, such as 2-methyl-3-furanthiol, 2-methyl-3-(methyldithio)furan, and 2-acetyl-2-thiazoline have already been detected in dry-cured hams. Additionally, the oxidative stability of hams can be enhanced by some B vitamins, owing to their antioxidant activity [33]. Here, B vitamins were significantly accumulated in mature DTP hams, indicating that fermentation could improve the antioxidant activity and nutritional value of DTP hams. This result was concordant with a previous report of high levels of B vitamins in Italian ham [31]. Therefore, the aging process exerted a great positive influence on the nutritional value of DTP hams, owing to the accumulation of essential amino acids, essential fatty acids, and vitamin B in the mature hams.

Four heterocyclic compounds were screened out in DTP ham. Pyridoxal decreased, while indole and indoleacetaldehyde (IAALD) increased along with the extension of fermentation time. Indole and IAALD are important heterocyclic systems which can be built into proteins of the amino acid tryptophan [31]. In our results, Figure 5B showed that indole and IAALD were also involved in the tryptophan metabolism pathway, and tryptophan transformation might indirectly lead to the increased contents of indole and IAALD in DTP hams. Zhu et al. [34] mentioned that tryptophan can be directly transformed into several molecules, including the ligands for the aromatic hydrocarbon receptor (AhR), such as indole, indole-3-aldehyde, and IAALD, with AhR signaling as a crucial part of the barrier site immune response for intestinal homeostasis.

Three nucleotides and their metabolites were screened out, and they were involved in the cofactor biosynthesis pathway. With increasing fermentation time, a decrease was observed in both adenosine-5′-monophosphate (AMP) and inosine-5′-monophosphate (IMP) levels, while levels of cytidine 5′-diphosphate increased first and then decreased, reaching their peak at 180 d. According to You and Luo [35], AMP, inosine, and hypoxanthine are the main nucleotide metabolites in boneless hams, with AMP converted to IMP by AMP deaminase, and IMP further degraded to hypoxanthine and inosine, suggesting an internal mutual transformation process for the changes in nucleotides and their metabolites, with these metabolites as down-stream compounds formed by ATP breakdown. However, inosine and hypoxanthine both contribute to the bitterness of hams and were not screened out in the DTP hams.

With increasing fermentation time, accumulation of 2-(formylamino) benzoic acid and 3-hydroxyanthranilic acid were observed, both of which are involved in tryptophan metabolism. As a metabolite of tryptophan metabolism, 3-hydroxyanthranilic acid can remove superoxide radicals and reduce oxidative damage [36]. The accumulation of 3-hydroxyanthranilic acid and vitamin B provides a possibility of antioxidant activity in mature DTP hams.

With increasing fermentation time, the DTP hams showed an increase in tryptamine. According to a previous report [37], no quantification has been performed for tryptamine in cured hams, and its high content in mature DTP hams might be attributed to the high content of tryptophan, the amine-precursor amino acid, whose content showed a time-dependent increase during ham maturation.

## 4. Conclusions

The evolution and transformation pathways of metabolites during DTP ham fermentation were explored by widely targeted metabonomic analysis. Metabolic spectral analysis identified 873 metabolites in 17 subclasses with significant changes in 448 differential metabolites in 16 categories identified, with the mature hams showing the highest levels of impact on amino acids and their metabolites, followed by nucleotides and their metabolites, GPs, organic acids and their derivatives, and FAs. KEGG enrichment analysis revealed the involvement of 65 key metabolites in the top 10 pathways, mainly including amino acids and their metabolites, FAs, and organic acids and their derivatives. Protein metabolism (2-oxocarboxylic acid metabolism, tryptophan metabolism, amino acid biosynthesis) and lipid metabolism (linoleic acid metabolism, unsaturated fatty acid biosynthesis) were shown to comprise the top five transformation pathways for metabolite markers in the mature hams. In summary, the unique taste, flavor, and nutritional value of mature DTP hams may be endowed by essential amino acids (L-lysine, L-threonine, L-tryptophan, L-phenylalanine, and L-valine), MSG-like amino acids (glutamic acid and L-aspartic), free fatty acids (AA, DHA, and EPA), citric acid, oxaloacetic acid, succinic acid, and vitamin B due to their significant accumulation in matured DTP hams. This study provides a better understanding of the evolution and transformation pathways of metabolites during DTP ham fermentation, providing useful information for the development of an innovative Diqing Tibetan pig industry.

## Data Availability

The raw data supporting the conclusions of this article will be made available by the authors on request.

## Supplementary Materials

The following supporting information can be downloaded at: https://www.mdpi.com/article/10.3390/foods14142468/s1, Table S1: The differential metabolites in Diqing Tibetan pig hams during fermentation, Figure S1: The KEGG pathway annotations from 448 differential metabolites. *X*-axis: the proportion and number of annotated metabolites; *Y*-axis: pathway.
