# Supplementary material for: Dynamic Changes in Metabolites and Transformation Pathways in Diqing Tibetan Pig Hams During Fermentation Determined by Widely Targeted Metabolomic Analysis
Source: Foods. 2025 Jul 14;14(14):2468. doi: 10.3390/foods14142468 (PMC12296140; doi:10.3390/foods14142468)
Supplement: Supplementary file 1 [file foods-14-02468-s001.zip › Supplementary data.pdf]

Table S1: The differential metabolites in Diqing Tibetan pig hams during fermentation

Class I	Class II	Compounds	Formula	P-value	VIP
Amino acid and Its metabolites	Amino acid derivatives	(R)-(-)-2-phenylglycine	C8H11NO	4.80E-32	1.32E+00
		1-Aminocyclobutanecarboxylic acid	C5H9NO2	4.65E-24	1.35E+00
		2,6-Diamino-5-hydroxyhexanoic acid	C6H14N2O3	1.15E-12	1.62E+00
		3-Bromotyrosine	C9H10BrNO3	7.72E-05	1.12E+00
		3-Hydroxy-L-phenylalanine	C9H11NO3	2.29E-22	1.32E+00
		5-Oxoproline	C5H7NO3	2.39E-25	1.28E+00
		Acetylvaline	C7H13NO3	3.48E-42	1.28E+00
		Cycloleucine	C6H11NO2	1.26E-26	1.38E+00
		Hexanoyl Glycine	C8H15NO3	6.11E-24	1.20E+00
		Kynurenic Acid	C10H7NO3	3.05E-40	1.36E+00
		L-Dopa	C9H11NO4	4.02E-39	1.32E+00
		L-Tryptophanamide	C11H13N3O	4.72E-28	1.35E+00
		L-kynurenine	C10H12N2O3	5.09E-38	1.21E+00
		L-threo-3-Methylaspartate	C5H9NO4	1.49E-21	1.31E+00
		Mycosporine-glycine	C10H15NO6	6.24E-06	1.08E+00
		N'-Formylkynurenine	C11H12N2O4	2.88E-24	1.18E+00
		N,N-Dimethylarginine	C8H18N4O2	3.40E-08	1.34E+00
		N-Acetyl-L-Glutamic Acid	C7H11NO5	1.36E-37	1.32E+00
		N-Acetyl-L-Histidine	C8H11N3O3	2.80E-27	1.26E+00
		N-Acetyl-L-Leucine	C8H15NO3	1.11E-46	1.28E+00
		N-Acetyl-L-Tyrosine	C11H13NO4	1.59E-41	1.17E+00
		N-Acetyl-L-alanine	C5H9NO3	5.29E-32	1.22E+00
		N-Acetyl-L-methionine	C7H13NO3S	4.15E-40	1.34E+00
		N-Acetyl-L-phenylalanine	C11H13NO3	9.63E-44	1.34E+00
		N-Acetylglycine	C4H7NO3	5.11E-24	1.01E+00
		N-Acetylthreonine	C6H11NO4	4.62E-08	1.39E+00
		N-Alpha-Acetyl-L-Asparagine	C6H10N2O4	2.60E-22	1.10E+00
		N-Amidino-L-Aspartate	C5H9N3O4	3.56E-28	1.00E+00
		N-Ethylglycine	C4H9NO2	5.75E-11	1.40E+00
		N-Methylalanine	C4H9NO2	5.75E-11	1.40E+00
		N-Myristoylglycine	C16H31NO3	1.03E-35	1.35E+00
		N-Palmitoylglycine	C18H35NO3	1.20E-22	1.18E+00
		N-acetyl-beta-alanine	C5H9NO3	9.25E-30	1.25E+00
		N-acetylornithine	C7H14N2O3	3.17E-30	1.03E+00
		N-glutarylglycine	C7H11NO5	2.91E-28	1.19E+00
		N-lactoyl-phenylalanine	C12H15NO4	1.67E-36	1.28E+00
		Nα-Acetyl-L-Arginine	C8H16N4O3	1.16E-17	1.06E+00
		Nα-Acetyl-L-glutamine	C7H12N2O4	9.26E-14	1.12E+00
		Phe-Pro	C14H18N2O3	8.86E-31	1.09E+00
		S-Allyl-L-cysteine	C6H11NO2S	3.66E-23	1.27E+00
		S-Methyl-L-Cysteine-S-oxide	C4H9NO3S	3.45E-11	1.26E+00
		S-Sulfo-L-Cysteine	C3H7NO5S2	2.43E-13	1.11E+00
	Amino acids	2-Octanamidoacetic acid	C10H19NO3	4.41E-38	1.35E+00
		D-Ornithine	C5H12N2O2	5.91E-41	1.47E+00
		L-Arginine	C6H14N4O2	2.93E-31	1.02E+00
		L-Aspartic Acid	C4H7NO4	1.29E-21	1.26E+00
		L-Cysteine	C3H7NO2S	2.91E-11	1.33E+00
		L-Glutamic Acid	C5H9NO4	1.49E-21	1.31E+00
		L-Glutamine	C5H10N2O3	7.03E-34	1.36E+00
		L-Glycine	C2H5NO2	4.08E-09	1.09E+00
		L-Homoarginine	C7H16N4O2	2.17E-20	1.11E+00
		L-Homocitrulline	C7H15N3O3	3.30E-21	1.20E+00
		L-Lysine	C6H14N2O2	6.22E-32	1.31E+00
		L-Methionine	C5H11NO2S	1.14E-02	1.25E+00
		L-Norleucine	C6H13NO2	2.86E-32	1.25E+00

	L-Norvaline	C5H11NO2	5.82E-29	1.32E+00
	L-Ornithine	C5H13ClN2O2	1.04E-34	1.51E+00
	L-Phenylalanine	C9H11NO2	5.01E-37	1.33E+00
	L-Proline	C5H9NO2	4.65E-24	1.35E+00
	L-Threonine	C4H9NO3	2.32E-09	1.23E+00
	L-Tryptophan	C11H12N2O2	3.18E-33	1.30E+00
	L-Tyrosine	C9H11NO3	6.82E-08	1.11E+00
	L-Valine	C5H11NO2	5.82E-29	1.32E+00
	N-Formylglycine	C3H5NO3	1.09E-10	1.36E+00
	N-Propionylglycine	C5H9NO3	1.19E-26	1.32E+00
	Pyroglutamic acid	C5H7NO3	4.98E-29	1.31E+00
	Sarcosine	C3H7NO2	7.01E-07	1.10E+00
	Ala-Met	C8H16N2O3S	2.97E-26	1.34E+00
	Ala-Phe	C12H16N2O3	2.74E-36	1.33E+00
	Arg-Asp	C10H19N5O5	1.24E-06	1.06E+00
	Arg-Leu	C12H25N5O3	2.71E-14	1.02E+00
	Arg-Met	C11H23N5O3S	3.83E-05	1.02E+00
	Arg-Phe	C15H23N5O3	5.53E-22	1.28E+00
	Arg-Val	C11H23N5O3	3.61E-13	1.41E+00
	Asn-Pro	C9H15N3O4	1.61E-18	1.15E+00
	Asp-Ile	C10H18N2O5	2.08E-32	1.20E+00
	Asp-Leu	C10H18N2O5	2.08E-32	1.20E+00
	Asp-Phe	C13H16N2O5	2.50E-31	1.22E+00
	Asp-Tyr	C13H16N2O6	7.17E-16	1.30E+00
	Asp-Val	C9H16N2O5	4.55E-19	1.22E+00
	Cyclo(Ala-Pro)	C8H12N2O2	1.65E-38	1.27E+00
	Cyclo(Phe-Glu)	C14H16N2O4	4.76E-40	1.34E+00
	Cyclo(Phe-Pro)	C14H16N2O2	4.58E-43	1.13E+00
	Cyclo(Pro-Glu)	C10H14N2O4	1.15E-37	1.30E+00
	Cyclo(Pro-Leu)	C11H18N2O2	5.42E-36	1.39E+00
	Cyclo(Pro-Phe)	C14H16N2O2	4.58E-43	1.13E+00
	Cyclo(Pro-Val)	C10H16N2O2	1.91E-38	1.42E+00
	Cyclo(Ser-Pro)	C8H12N2O3	8.53E-38	1.25E+00
	Cys-Gly	C5H10N2O3S	2.18E-13	1.07E+00
	Glu-Arg	C11H21N5O5	8.39E-22	1.35E+00
Small Peptide	Glu-Glu-Ile	C16H27N3O8	2.17E-25	1.08E+00
	Glu-Ile	C11H20N2O5	2.50E-11	1.17E+00
	Glu-Leu	C11H20N2O5	6.10E-39	1.20E+00
	Glu-Met	C10H18N2O5S	1.31E-41	1.25E+00
	Glu-Phe	C14H18N2O5	6.76E-37	1.19E+00
	Glu-Pro	C10H16N2O5	1.22E-36	1.29E+00
	Glu-Ser	C8H14N2O6	2.36E-27	1.11E+00
	Glu-Thr	C9H16N2O6	7.78E-37	1.32E+00
	Glu-Tyr	C14H18N2O6	1.87E-30	1.36E+00
	Glu-Val	C10H18N2O5	9.39E-33	1.18E+00
	Glutathione Oxidized	C20H32N6O12S2	3.17E-05	1.23E+00
	Glutathione Reducedform	C10H17N3O6S	6.65E-13	1.01E+00
	Gly-Gly-Phe	C13H17N3O4	2.63E-29	1.13E+00
	Gly-Val	C7H14N2O3	6.54E-11	1.14E+00
	His-Val	C11H18N4O3	9.08E-24	1.04E+00
	Hyp-Thr	C9H16N2O5	1.08E-07	1.28E+00
	Ile-Arg	C12H25N5O3	3.24E-09	1.10E+00
	Ile-Asn	C10H19N3O4	4.67E-06	1.27E+00
	Ile-Asp	C10H18N2O5	2.08E-32	1.20E+00
	Ile-Gln	C11H21N3O4	2.69E-07	1.08E+00
	Ile-Glu	C11H20N2O5	2.50E-11	1.17E+00
	Ile-Leu	C12H24N2O3	9.31E-28	1.09E+00
	Ile-Met	C11H22N2O3S1	1.18E-40	1.18E+00

Ile-Ser	C9H18N2O4	2.19E-03	1.29E+00
Ile-Thr	C10H20N2O4	3.52E-08	1.04E+00
Ile-Trp	C17H23N3O3	1.74E-30	1.19E+00
Ile-Tyr	C15H22N2O4	1.17E-29	1.33E+00
Ile-Val	C11H22N2O3	2.82E-32	1.28E+00
Leu-Arg	C12H25N5O3	1.11E-19	1.21E+00
Leu-Asp	C10H18N2O5	9.84E-07	1.34E+00
Leu-Glu	C11H20N2O5	2.50E-11	1.17E+00
Leu-Leu	C12H24N2O3	9.31E-28	1.09E+00
Leu-Met	C11H22N2O3S	5.12E-31	1.10E+00
Leu-Phe	C15H22N2O3	8.30E-35	1.11E+00
Leu-Thr	C10H20N2O4	3.52E-08	1.04E+00
Leu-Trp	C17H23N3O3	1.74E-30	1.19E+00
Leu-Tyr	C15H22N2O4	1.17E-29	1.33E+00
Leu-Val	C11H22N2O3	1.20E-37	1.22E+00
Lys-Ser	C9H19N3O4	2.24E-27	1.05E+00
Lys-Thr	C10H21N3O4	8.54E-17	1.33E+00
Met-Glu	C10H18N2O5S	1.62E-40	1.27E+00
Met-Met	C10H20N2O3S2	1.77E-36	1.15E+00
Met-Tyr	C14H20N2O4S	5.48E-35	1.10E+00
Met-Val	C10H20N2O3S	2.73E-32	1.34E+00
N(Alpha)-Acetyl-Epsilon-(2-Propenal)Lysine	C11H18N2O4	4.78E-38	1.35E+00
Phe-Glu	C14H18N2O5	2.33E-42	1.19E+00
Phe-Gly	C11H14N2O3	1.80E-22	1.17E+00
Phe-Met	C14H20N2O3S	5.84E-29	1.33E+00
Phe-Phe	C18H20N2O3	2.81E-38	1.20E+00
Phe-Ser	C12H16N2O4	6.21E-35	1.23E+00
Phe-Thr	C13H18N2O4	1.05E-37	1.06E+00
Phe-Tyr	C18H20N2O4	3.69E-39	1.08E+00
Phe-Val	C14H20N2O3	1.98E-35	1.12E+00
Pro-Asn	C9H15N3O4	4.47E-16	1.30E+00
Pro-Gly	C7H12N2O3	6.31E-15	1.09E+00
Pro-His	C11H16N4O3	1.66E-14	1.23E+00
Pro-Ile	C11H20N2O3	7.53E-40	1.36E+00
Pro-Leu	C11H20N2O3	8.95E-35	1.11E+00
Pro-Met	C10H18N2O3S	4.67E-24	1.05E+00
Pro-Ser	C8H14N2O4	9.13E-06	1.38E+00
Pro-Trp	C16H19N3O3	2.82E-29	1.28E+00
Pro-Val	C10H18N2O3	5.67E-17	1.04E+00
Proline-Hydroxyproline	C10H16N2O4	1.67E-38	1.11E+00
Ser-Ile	C9H18N2O4	4.67E-35	1.15E+00
Ser-Leu	C9H18N2O4	1.47E-35	1.16E+00
Ser-Lys	C9H19N3O4	9.84E-31	1.15E+00
Ser-Phe	C12H16N2O4	2.09E-34	1.10E+00
Ser-Pro	C8H14N2O4	3.60E-08	1.26E+00
Ser-Trp	C14H17N3O4	1.53E-29	1.25E+00
Thr-Phe	C13H18N2O4	1.05E-37	1.06E+00
Trp-Gly	C13H15N3O3	9.06E-35	1.21E+00
Trp-Val	C16H21N3O3	3.47E-33	1.21E+00
Tyr-Ile	C15H22N2O4	8.92E-30	1.22E+00
Tyr-Leu	C15H22N2O4	8.40E-39	1.10E+00
Tyr-Pro	C14H18N2O4	1.75E-37	1.18E+00
Tyr-Tyr	C18H20N2O5	1.09E-38	1.34E+00
Tyr-Val	C14H20N2O4	1.29E-32	1.10E+00
Val-Asp	C9H16N2O5	6.65E-04	1.20E+00
Val-Glu	C10H18N2O5	4.45E-18	1.15E+00
Val-His	C11H18N4O3	6.63E-22	1.14E+00
Val-Ile	C11H22N2O3	1.71E-31	1.31E+00

		Val-Leu	C11H22N2O3	1.71E-31	1.31E+00
		Val-Met	C10H20N2O3S	4.93E-13	1.21E+00
		Val-Phe-Ala	C17H25N3O4	1.30E-23	1.37E+00
		Val-Trp	C16H21N3O3	1.30E-32	1.19E+00
		Val-Val	C10H20N2O3	7.58E-25	1.25E+00
		cyclo(Pro-Tyr)	C14H16N2O3	3.01E-31	1.09E+00
		cyclo(glu-glu)	C10H14N2O6	1.09E-36	1.28E+00
		cyclo(gly-pro)	C7H10N2O2	1.01E-38	1.24E+00
		cyclo(pro-pro)	C10H14N2O2	8.28E-41	1.33E+00
		gamma-Glu-Ala	C8H14N2O5	5.37E-06	1.20E+00
		γ-Glu-Met	C10H18N2O5S	2.59E-23	1.27E+00
Nucleotide and Its metabolites	Nucleotide and Its metabolites	1-Methylguanine	C6H7N5O	7.39E-28	1.32E+00
		1-Methylguanosine	C11H15N5O5	2.86E-08	1.25E+00
		1-Methylinosine	C11H14N4O5	8.78E-16	1.31E+00
		2'-Aenylic Acid	C10H14N5O7P	3.03E-14	1.32E+00
		2'-Deoxyinosine-5'-monophosphate	C10H13N4O7P	6.91E-21	1.28E+00
		2'-Deoxyuridine	C9H12N2O5	8.43E-25	1.06E+00
		2'-O-Methylguanosine	C11H15N5O5	1.35E-20	1.47E+00
		2'-O-methyladenosine	C11H15N5O4	1.73E-13	1.50E+00
		2'-O-methyluridine	C10H14N2O6	1.30E-29	1.46E+00
		2-(Dimethylamino)Guanosine	C12H17N5O5	1.93E-29	1.43E+00
		2-Aminomethylpyrimidine	C5H7N3	2.19E-12	1.02E+00
		2-Hydroxy-6-Aminopurine	C5H5N5O	8.20E-09	1.49E+00
		2-Hydroxyadenosine	C10H13N5O5	1.69E-06	1.09E+00
		2-Methylguanosine	C11H15N5O5	5.21E-13	1.45E+00
		3'-Adenylic acid	C10H14N5O7P	8.83E-24	1.55E+00
		3-Methyluridine	C10H14N2O6	2.02E-13	1.24E+00
		5'-Deoxy-5'-(Methylthio) Adenosine	C11H15N5O3S	3.04E-24	1.11E+00
		5,6-Dihydrouridine	C9H14N2O6	8.78E-07	1.35E+00
		5-Acetylamino-6-amino-3-methyluracil	C7H10N4O3	2.40E-23	1.30E+00
		5-Hydroxy-2'-deoxyuridine	C9H12N2O6	1.03E-09	1.42E+00
		5-Methylcytosine	C5H7N3O	1.31E-07	1.33E+00
		5-Methyluridine	C10H14N2O6	3.94E-30	1.43E+00
		6-O-methylguanine	C6H7N5O	7.39E-28	1.32E+00
		7-Methylguanine	C6H7N5O	1.04E-19	1.22E+00
		7-Methylguanosine	C11H15N5O5	2.86E-08	1.25E+00
		7-Methylxanthine	C6H6N4O2	2.44E-06	1.42E+00
		8-Azaguanine	C4H4N6O	1.19E-16	1.23E+00
		8-Hydroxyguanosine	C10H13N5O6	1.70E-20	1.20E+00
		Adenosine	C10H13N5O4	2.54E-22	1.26E+00
		Adenosine 5'-Monophosphate	C10H14N5O7P	2.94E-13	1.17E+00
		Cyclic ADP ribose	C15H21N5O13P2	5.82E-14	1.36E+00
		Cytidine	C9H13N3O5	2.58E-20	1.11E+00
		Cytidine 5'-Diphosphocholine	C14H26N4O11P2	3.57E-12	1.22E+00
		Cytidine 5'-diphosphate	C9H15N3O11P2	6.37E-05	1.19E+00
		Deoxycytidine	C9H13N3O4	6.29E-14	1.35E+00
		Deoxyguanosine 5'-monophosphate(dGMP)	C10H14N5O7P	1.29E-11	1.22E+00
		Guanine	C5H5N5O	2.78E-20	1.45E+00
		Guanosine	C10H13N5O5	4.01E-23	1.31E+00
		Inosine 5'-monophosphate	C10H13N4O8P	1.37E-30	1.46E+00
		Inosine diphosphate	C10H14N4O11P2	1.95E-12	1.02E+00
		N6-(2-Hydroxyethyl)adenosine	C12H17N5O5	3.39E-27	1.39E+00
		N6-Isopentenyladenosine	C15H21N5O4	5.13E-30	1.35E+00
		N6-isopentene adenine	C10H13N5	2.41E-29	1.36E+00
		Oxypurinol	C5H4N4O2	1.19E-16	1.23E+00
		Purine	C5H4N4	5.78E-33	1.32E+00
		Ribosyladenosine	C15H21N5O8	1.24E-19	1.14E+00
		Theobromine	C7H8N4O2	6.51E-26	1.29E+00

		Thymidine	C10H14N2O5	4.79E-23	1.36E+00
		Thymine	C5H6N2O2	9.47E-45	1.43E+00
		Xanthine	C5H4N4O2	3.91E-22	1.38E+00
		Xanthosine	C10H12N4O6	6.38E-06	1.45E+00
FA	CAR	Carnitine C3:0	C10H19NO4	4.09E-27	1.24E+00
		Carnitine C4:0	C11H21NO4	1.33E-32	1.08E+00
		Carnitine C5:1	C12H21NO4	2.96E-21	1.22E+00
		Carnitine C6:0	C13H25NO4	5.80E-18	1.27E+00
		Carnitine C7:0	C14H27NO4	3.67E-21	1.18E+00
		Carnitine C9:0	C16H31NO4	1.27E-21	1.44E+00
		Carnitine isoC4:0	C11H21NO4	1.33E-32	1.08E+00
	FFA	8,15-Dihete	C20H32O4	1.87E-17	1.28E+00
		AA	C20H32O2	1.89E-33	1.39E+00
		DHA	C22H32O2	3.32E-24	1.32E+00
		DL-2-hydroxystearic acid	C18H36O3	1.36E-02	1.19E+00
		EPA	C20H30O2	2.40E-27	1.34E+00
		FFA(16:0)	C16H32O2	5.15E-14	1.38E+00
		FFA(16:1)	C16H30O2	2.67E-32	1.33E+00
		FFA(18:1)	C18H34O2	3.02E-36	1.28E+00
		FFA(18:2)	C18H32O2	1.88E-30	1.32E+00
		FFA(18:3)	C18H30O2	1.15E-09	1.30E+00
		FFA(18:4)	C18H28O2	7.29E-29	1.32E+00
		FFA(20:2)	C20H36O2	6.59E-25	1.30E+00
		FFA(20:4)	C20H32O2	7.79E-31	1.38E+00
		FFA(22:4)	C22H36O2	1.29E-27	1.26E+00
		FFA(22:6)	C22H32O2	4.34E-26	1.32E+00
		Pinolenic acid	C18H30O2	9.21E-21	1.30E+00
	Oxidized lipids	(±)12-HETE	C20H32O3	2.69E-31	1.32E+00
		(±)15-HETE	C20H32O3	2.97E-29	1.31E+00
		(±)5-HEPE	C20H30O3	2.85E-25	1.29E+00
		(±)5-HETE	C20H32O3	1.40E-27	1.30E+00
		(±)9-HETE	C20H32O3	1.40E-27	1.30E+00
		11,12-EET	C20H32O3	4.35E-33	1.34E+00
		11-HEDE	C20H36O3	9.57E-31	1.13E+00
		12,13-DiHOME	C18H34O4	2.72E-32	1.03E+00
		12,13-EpOME	C18H32O3	4.71E-35	1.19E+00
		13(R)-HODE	C18H32O3	8.98E-33	1.06E+00
		13-oxoODE	C18H30O3	1.00E-15	1.32E+00
		15(S)-HETrE	C20H34O3	1.16E-28	1.29E+00
		15-HEDE	C20H36O3	9.57E-31	1.13E+00
		15-oxoETE	C20H30O3	9.80E-33	1.30E+00
		5-HETrE	C20H34O3	1.16E-28	1.29E+00
		5-oxoETE	C20H30O3	4.53E-26	1.29E+00
		8(S)-HETrE	C20H34O3	1.16E-28	1.29E+00
		9(S),12(S),13(S)-TriHOME	C18H34O5	1.62E-36	1.08E+00
		9,10-DiHOME	C18H34O4	1.29E-39	1.16E+00
		9,10-EpOME	C18H32O3	4.71E-35	1.19E+00
		9-oxoODE	C18H30O3	1.00E-15	1.32E+00
		PGF3α	C20H32O5	1.23E-20	1.22E+00
		Tetranor-12(R)-HETE	C16H26O3	2.97E-04	1.29E+00
	Others	Docosahexaenoic Acid Glycine	C24H35NO3	1.75E-38	1.39E+00
		Methanandamide	C23H39NO2	4.52E-40	1.25E+00
		N-Arachidonic-acid-L-serine	C23H37NO4	7.47E-33	1.25E+00
		N-Arachidonoyl-L-Alanine	C23H37NO3	1.61E-38	1.40E+00
GP	LPC	LPC(0:0/15:0)	C23H48NO7P	4.96E-15	1.23E+00
		LPC(0:0/16:0)	C24H50NO7P	4.72E-13	1.24E+00
		LPC(0:0/18:0)	C26H54NO7P	1.04E-03	1.07E+00

		LPC(0:0/18:1)	C26H52NO7P	6.23E-16	1.05E+00
		LPC(0:0/18:2)	C26H50NO7P	7.83E-24	1.86E+00
		LPC(0:0/20:2)	C28H54NO7P	6.38E-29	1.62E+00
		LPC(0:0/20:4)	C28H50NO7P	8.98E-26	1.61E+00
		LPC(15:0/0:0)	C23H48NO7P	4.96E-15	1.23E+00
		LPC(16:0/0:0)	C24H50NO7P	4.72E-13	1.24E+00
		LPC(18:0/0:0)	C26H54NO7P	1.04E-03	1.07E+00
		LPC(18:1/0:0)	C26H52NO7P	6.23E-16	1.05E+00
		LPC(18:2/0:0)	C26H50NO7P	7.83E-24	1.86E+00
		LPC(20:2/0:0)	C28H54NO7P	6.38E-29	1.62E+00
		LPC(20:4/0:0)	C28H50NO7P	8.98E-26	1.61E+00
		LPC(22:5/0:0)	C30H52NO7P	2.93E-20	1.78E+00
		LPC(O-0:0/18:0)	C26H56NO6P	1.75E-05	1.29E+00
		LPC(O-16:0/2:0)	C26H54NO7P	1.04E-03	1.07E+00
		LPC(O-18:0/0:0)	C26H56NO6P	1.75E-05	1.29E+00
		LPC(O-18:1/0:0)	C26H54NO6P	4.40E-27	1.07E+00
	LPE	LPE(0:0/16:0)	C21H44NO7P	3.26E-10	1.56E+00
		LPE(0:0/16:1)	C21H42NO7P	5.43E-04	1.05E+00
		LPE(0:0/18:0)	C23H48NO7P	5.80E-04	1.40E+00
		LPE(0:0/20:2)	C25H48NO7P	3.40E-08	1.43E+00
		LPE(0:0/22:6)	C27H44NO7P	1.91E-28	1.14E+00
		LPE(16:0/0:0)	C21H44NO7P	3.26E-10	1.56E+00
		LPE(18:0/0:0)	C23H48NO7P	5.80E-04	1.40E+00
		LPE(18:1/0:0)	C23H46NO7P	9.69E-06	1.44E+00
		LPE(20:2/0:0)	C25H48NO7P	3.40E-08	1.43E+00
		LPE(22:6/0:0)	C27H44NO7P	1.91E-28	1.14E+00
		Phosphatidylethanolamine lyso alkenyl 16:0	C21H44NO6P	1.60E-08	1.32E+00
		Phosphatidylethanolamine lyso alkenyl 18:2	C23H44NO6P	1.52E-29	1.28E+00
	PC O	PC(O-16:0/0:0)	C24H52NO6P	5.06E-22	1.10E+00
		PC(O-16:0/O-1:0)	C25H54NO6P	2.03E-12	1.23E+00
		PC(O-16:0/O-2:0)	C26H56NO6P	1.75E-05	1.29E+00
	PE	O-Phosphorylethanolamine	C2H8NO4P	1.70E-19	1.15E+00
Organic acid and Its derivatives	Organic acid and Its derivatives	(R)-2-Hydroxybutyric acid	C4H8O3	3.79E-07	1.18E+00
		(R)-3-Hydroxymyristic acid	C14H28O3	2.37E-20	1.28E+00
		1-Aminocyclohexanecarboxylic acid	C7H13NO2	1.34E-31	1.35E+00
		2,6-Diaminopimelic acid	C7H14N2O4	2.33E-08	1.05E+00
		2-Methyllactic acid	C4H8O3	1.14E-06	1.19E+00
		2-amino-4-oxovaleric acid	C5H9NO3	5.29E-32	1.22E+00
		2-methyl citric acid	C7H10O7	3.07E-18	1.11E+00
		3,5-Dimethoxy-4-Hydroxycinnamic Acid	C11H12O5	5.21E-27	1.18E+00
		3-Amino-4-methylpentanoic acid	C6H13NO2	1.14E-28	1.28E+00
		3-Amino-5-hydroxybenzoic acid	C7H7NO3	3.29E-21	1.14E+00
		3-Dehydroshikimate	C7H8O5	2.33E-20	1.09E+00
		3-Hydroxy-tetradecanoic?acid	C14H28O3	2.37E-20	1.28E+00
		4-Acetylaminobenzoic acid	C9H9NO3	7.95E-29	1.29E+00
		Allantoin	C4H6N4O3	8.27E-16	1.22E+00
		Citric Acid	C6H8O7	9.45E-27	1.26E+00
		D-Malic acid	C4H6O5	7.90E-09	1.36E+00
		Dihydrocaffeic acid	C9H10O4	3.98E-20	1.22E+00
		Hippuric Acid	C9H9NO3	7.95E-29	1.29E+00
		L-2-amino-6-oximelic acid	C7H11NO5	9.80E-34	1.31E+00
		L-Lactic Acid	C3H6O3	4.58E-04	1.04E+00
		Maleic Acid	C4H4O4	4.56E-10	1.30E+00
		Methylmalonic Acid	C4H6O4	1.54E-16	1.02E+00
		Oxaloacetic acid	C4H4O5	3.48E-28	1.06E+00
		Piperidine acid	C6H11NO2	7.89E-29	1.24E+00
		SDMA	C8H18N4O2	1.31E-13	1.30E+00

		Succinic Acid	C4H6O4	1.54E-16	1.02E+00
		Ureidoisobutyric Acid	C5H10N2O3	8.62E-38	1.30E+00
	Phosphoric acids	5-O-(1-carboxyvinyl)-3-phosphate	C10H13O10P	9.08E-14	1.19E+00
		Carbamoyl phosphate	CH4NO5P	1.70E-19	1.15E+00
		Glycerol 3-Phosphate	C3H9O6P	7.05E-05	1.39E+00
		Phosphoenolpyruvate	C3H5O6P	1.83E-22	1.36E+00
		Phosphonoacetic acid	C2H5O5P	6.84E-32	1.38E+00
	Sulfonic acids	Guanidinoethyl Sulfonate	C3H9N3O3S	1.19E-12	1.04E+00
		Isethionic acid	C2H6O4S	3.56E-11	1.00E+00
		N-2-hydroxyethylpiperazine-N''-2-ethanesulfonic acid	C8H18N2O4S	2.79E-13	1.12E+00
Heterocyclic compounds	Heterocyclic compounds	1-pyrroline-4-hydroxy-2-carboxylate	C5H7NO3	1.83E-08	1.35E+00
		2-Methyl-1-Pyrroline	C5H9N	1.64E-26	1.35E+00
		3-Amino-2-piperidinone	C5H10N2O	5.05E-20	1.42E+00
		4-Aminomethylpyrimidine	C5H7N3	3.03E-21	1.24E+00
		5,6-Dimethylbenzimidazole	C9H10N2	2.97E-20	1.34E+00
		Caffeine	C8H10N4O2	1.11E-11	1.53E+00
		Hydroxyquinoline	C9H7NO	3.00E-36	1.26E+00
		Lumichrome	C12H10N4O2	5.57E-16	1.00E+00
		N-(2-hydroxyethyl)-3-pyridinecarboxamide	C8H10N2O2	4.44E-33	1.33E+00
		Piperazine	C4H10N2	1.89E-21	1.09E+00
		Pyrrolidine	C4H9N	8.42E-21	1.26E+00
	Indole and Its derivatives	1-acetylindole	C10H9NO	4.58E-26	1.34E+00
		Ethyl 3-Indoleacetate	C12H13NO2	5.72E-20	1.01E+00
		Imidazoleacetic acid	C5H6N2O2	1.94E-13	1.02E+00
		Indole	C8H7N	8.38E-28	1.17E+00
		Indole 3-carbinol	C9H9NO	1.72E-27	1.07E+00
		Indole-3-Carboxaldehyde	C9H7NO	8.36E-27	1.07E+00
		Indole-3-carboxylic acid	C9H7NO2	2.89E-17	1.31E+00
		Indole-4-carboxaldehyde	C9H7NO	8.36E-27	1.07E+00
		Indoleacetaldehyde	C10H9NO	1.07E-30	1.34E+00
	Pteridines and derivatives	2,4-Dihydroxypteridine	C6H4N4O2	1.13E-13	1.08E+00
		4-Pyridoxic Acid	C8H9NO4	5.38E-32	1.15E+00
		L-Sepiapterin	C9H11N5O3	3.66E-39	1.23E+00
		Pyridoxal	C8H9NO3	1.27E-14	1.03E+00
Benzene and substituted derivatives	Benzene and substituted derivatives	1-Hydroxylamino-2-phenylethane	C8H11NO	4.80E-32	1.32E+00
		2',4'-Dihydroxyacetophenone	C8H8O3	4.07E-18	1.03E+00
		2-Phenylpropylamine	C9H13N	2.27E-25	1.15E+00
		3-Aminoquinoline	C9H8N2	1.65E-26	1.34E+00
		3-Chloroaniline	C6H6ClN	2.25E-09	1.34E+00
		3-aminobenzamide	C7H8N2O	1.28E-17	1.04E+00
		4,4'-Methylenedianiline	C13H14N2	3.64E-26	1.35E+00
		4-Hydroxybenzyl alcohol	C7H8O2	2.14E-09	1.16E+00
		Benzaldehyde	C7H6O	1.14E-31	1.33E+00
		GSK 4716	C17H18N2O2	8.82E-06	1.19E+00
	Phenolic acids	2-(Formylamino)Benzoic Acid	C8H7NO3	2.25E-06	1.12E+00
		2-Hydroxycinnamic acid	C9H8O3	1.42E-02	1.06E+00
		3-Amino-4-Hydroxybenzoic Acid	C7H7NO3	3.29E-21	1.14E+00
		3-Hydroxyanthranilic Acid	C7H7NO3	3.29E-21	1.14E+00
		Methylparaben	C8H8O3	4.07E-18	1.03E+00
		P-Coumaric Acid	C9H8O3	3.62E-15	1.32E+00
	Phenolics	4-acetoxyphenol	C8H8O3	1.11E-30	1.38E+00
		Hydroumbellic acid	C9H10O4	9.90E-20	1.11E+00
Carbohydrates and Its metabolites	Phosphate sugars	2-Deoxyribose 1-Phosphate	C5H11O7P	9.86E-19	1.31E+00
		2-Methyl-d-erythritol 2,4-cyclodiphosphate	C5H12O9P2	1.38E-10	1.13E+00
		Sucrose 6′-monophosphate	C12H23O14P	2.92E-13	1.37E+00
	Sugar alcohols	D-Arabitol	C5H12O5	2.56E-12	1.21E+00

		D-Mannitol	C6H14O6	7.34E-20	1.11E+00
		D-Sorbitol	C6H14O6	7.34E-20	1.11E+00
		GalactinolHydrate	C12H22O11	3.86E-11	1.09E+00
		Ribitol	C5H12O5	2.56E-12	1.21E+00
	Sugar derivatives	2,4-diacetamino-2,4,6-triphenoxy-D-mannopyranose	C10H18N2O5	9.32E-37	1.03E+00
	Sugars	D-Trehalose	C12H22O11	2.04E-03	1.07E+00
		D-ribonate lithium salt	C5H10O6	6.09E-33	1.00E+00
		Erythrose	C4H8O4	4.89E-12	1.05E+00
		L-Erythrulose	C4H8O4	4.89E-12	1.05E+00
		Lactose	C12H22O11	2.04E-03	1.07E+00
		Lactulose	C12H22O11	2.04E-03	1.07E+00
		Maltose	C12H22O11	2.04E-03	1.07E+00
		Melibiose	C12H22O11	1.27E-12	1.17E+00
		Stachyose	C24H42O21	3.15E-16	1.35E+00
Alcohol and amines	Alcohols	2-Methyl-5-nitroimidazole-1-ethanol	C6H9N3O3	7.90E-45	1.11E+00
		Triethanolamine	C6H15NO3	2.95E-10	1.09E+00
	Amines	(R)-(-)-1-Amino-2-propanol	C3H9NO	3.76E-07	1.23E+00
		2-(Acetylamino)-2-deoxy-A-D-glucopyranose	C8H15NO6	7.32E-06	1.30E+00
		2-(α-D-mannosyl)-3-phosphate glyceride	C9H17O12P	8.06E-38	1.45E+00
		5-Methoxytryptamine	C11H14N2O	1.51E-25	1.34E+00
		Acrylamide	C3H5NO	1.34E-31	1.30E+00
		N-(2-hydroxyethyl)stearamide	C20H41NO2	1.53E-05	1.30E+00
		N-Acetylhistamine	C7H11N3O	3.44E-08	1.39E+00
		N-Oleoyl Glycine	C20H37NO3	7.05E-26	1.52E+00
		Palmitoyl-EA	C18H35NO2	2.64E-28	1.28E+00
		Palmitoylethanolamide（PEA）	C18H37NO2	1.22E-26	1.49E+00
	Polyamines	Biotinamide	C10H17N3O2S	3.45E-21	1.19E+00
		Guanidine	CH5N3	1.81E-14	1.30E+00
		Hypotaurocyamine	C3H9N3O2S	1.10E-19	1.37E+00
		Oxidopamine	C8H11NO3	1.46E-11	1.25E+00
		Spermidine	C7H19N3	9.09E-10	1.20E+00
CoEnzyme and vitamins	CoEnzyme and vitamins	L-Ascorbate	C6H8O6	1.20E-04	1.21E+00
		Nicotinuric Acid	C8H8N2O3	3.47E-25	1.30E+00
		Pantothenate	C9H17NO5	1.40E-16	1.08E+00
		Pantothenol	C9H19NO4	1.42E-13	1.27E+00
		Pyridoxine	C8H11NO3	1.80E-14	1.37E+00
		Riboflavin	C17H20N4O6	4.18E-29	1.28E+00
		Thiamine	C12H17N4OS+	5.76E-17	1.20E+00
Bile acids	Bile acids	Glycohyodeoxycholic acid	C26H43NO5	1.22E-18	1.06E+00
		Tauro-beta-muricholic acid	C26H45NO7S	7.93E-09	1.18E+00
		Taurohyocholic acid	C26H45NO7S	7.93E-09	1.18E+00
Aldehyde, Ketones, Esters	Esters	D-Erythronolactone	C4H6O4	2.29E-03	1.21E+00
		N-arachidene glycine	C22H35NO3	7.39E-38	1.40E+00
SL	SM	Dihydro-D-sphingosine	C18H39NO2	6.20E-04	1.03E+00
		Sphingosyl-phosphocholine	C23H50N2O5P	4.41E-35	1.02E+00
Tryptamines, Cholines, Pigments	Cholines	Choline	C5H14NO+	7.61E-15	1.36E+00
	Tryptamines	Tryptamine	C10H12N2	9.00E-30	1.34E+00
Hormones and hormone related compounds	Hormones and hormone related compounds	20,26-dihydroxyecdysone	C27H44O8	1.08E-13	1.27E+00
Others	Others	Hydroxyurea	CH4N2O2	8.20E-09	1.36E+00

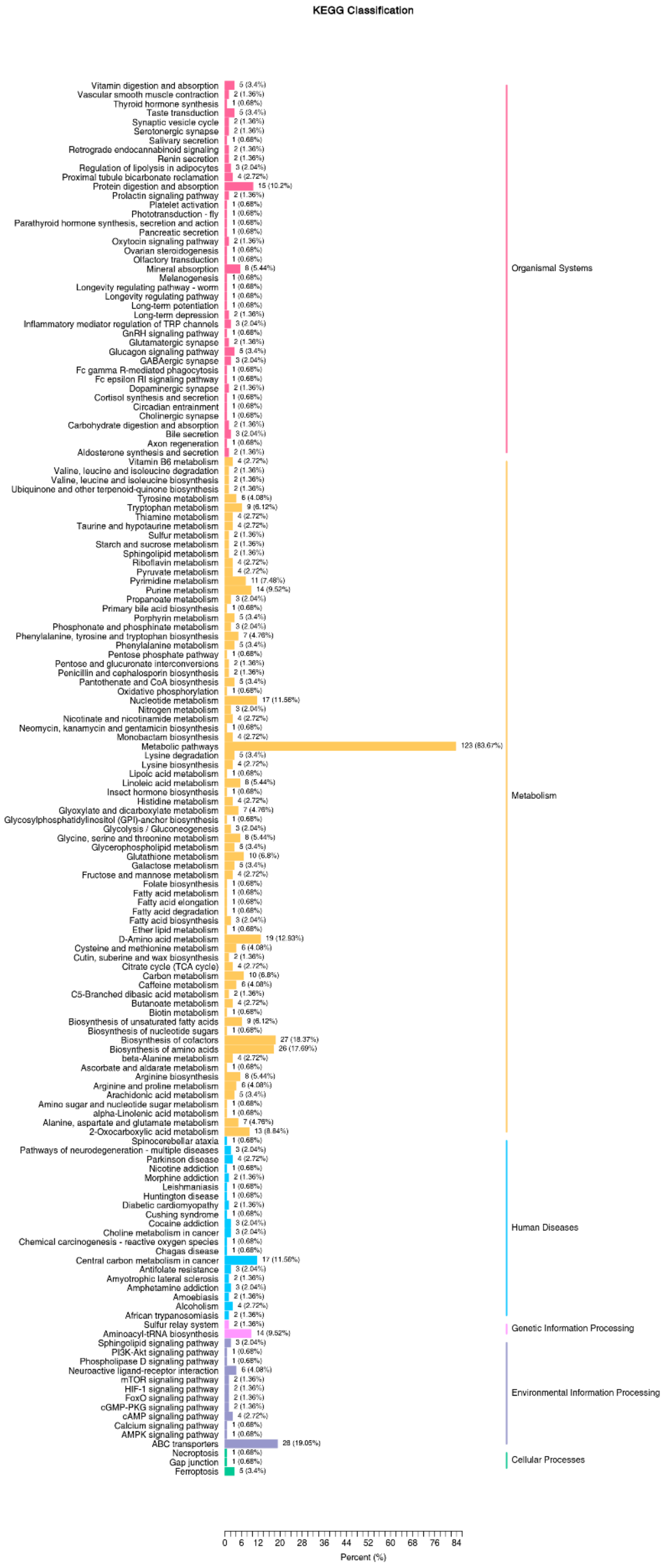

Figure S1: The KEGG pathway annotations from 448 differential metabolites. X-axis: the proportion and number of annotated metabolites; Y-axis: pathway.
